# Exploring the nutritional and biological properties of green coffee extracts: A comparative study of aqueous and enzymatic extraction processes

**DOI:** 10.1016/j.crfs.2024.100890

**Published:** 2024-10-29

**Authors:** Flávia Souza Almeida, Fernanda Furlan Goncalves Dias, Matthew William Ford, Stanislau Bogusz Junior, Ana Carla Kawazoe Sato, Juliana Maria Leite Nobrega de Moura Bell

**Affiliations:** a Department of Food Science and Technology, University of California, Davis, One Shields Avenue, Davis, CA, 95616, United States; b Department of Food Engineering and Technology, School of Food Engineering, University of Campinas (UNICAMP), 13083-862, Campinas, SP, Brazil; c University of São Paulo (USP), São Carlos Institute of Chemistry (IQSC), 13566-590, São Carlos, SP, Brazil; d Biological and Agricultural Engineering, University of California, One Shields Avenue, Davis, CA, 95616, United States

**Keywords:** Phenolic compounds, Protein hydrolysate, Angiotensin-converting enzyme, Lipase, α-glucosidase, Antioxidant activity, Protein digestibility

## Abstract

The effects of aqueous (AEP) and enzyme-assisted aqueous extraction processes (EAEP) on the biological and nutritional properties of green coffee extracts (protein and antioxidant-rich fraction) were investigated. All extracts exhibited high *in vitro* protein digestibility (>98%), regardless of the pH and use of enzymes during extraction, probably due to the low molecular weight of coffee proteins. Raising extraction pH from 7.0 to 9.0 resulted in extracts with lower concentrations of caffeine and some phenolic compounds such as chlorogenic and cinnamic acids, as well as catechin and epicatechin. This led to a reduction in the antioxidant activity of the extracts obtained at alkaline pH (AEP – pH 9.0). Overall, higher phenolic and caffeine extractability was achieved at neutral pH (AEP – pH 7.0), with no observed improvement in extraction yields when carbohydrases and/or proteases were employed. Coffee extracts generated by AEP at pH 7.0 exhibited the highest lipase inhibitory activity (66%), primarily attributed to their higher chlorogenic acid concentration. Conversely, EAEP extracts exhibited higher angiotensin-converting enzyme inhibition (up to 85%) compared to AEP extracts (68.5–74.3%). This strong inhibitory activity is likely related to the presence of both phenolic compounds (mainly chlorogenic acid) and smaller peptides. Nevertheless, all extracts exhibited low effectiveness for α-glucosidase inhibition (≤14%) and antimicrobial activity against *S. aureus* and *E. coli*. The current research underscores the feasibility of modulating the composition of green coffee extracts using sustainable and scalable AEP and EAEP, paving the way for developing tailored extracts with specific biological properties.

## Highlights

• Green coffee bean extracts are rich sources of bioactive compounds.
• Alkaline extraction reduced the concentration of phenolic compounds and caffeine.
• Proteolysis resulted in extracts with higher ACE inhibitory activity than aqueous treatments.
• Aqueous extracts produced at pH 7 exhibited highest lipase inhibitory activity.
• Coffee extracts presented limited antimicrobial activity and α-glucosidase inhibition.

## Graphical abstract

**Figure:**
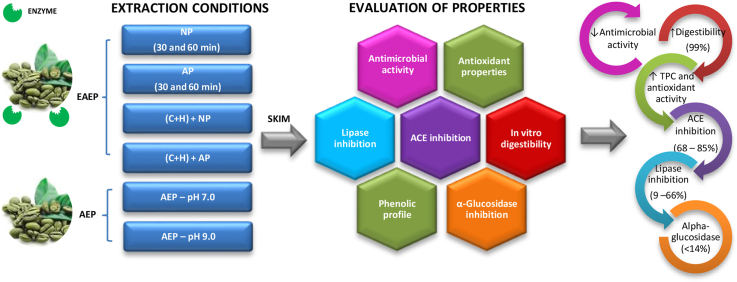

## 1 Introduction

Coffee beans serve not only as the foundation for producing the second most popular beverage in the world (Gaascht et al., 2015) but also represent a rich source of macronutrients such as carbohydrates, lipids, proteins, and micronutrients such as minerals. Additionally, they contain minor components such as caffeine and chlorogenic acids that contribute to the prevention of oxidative stress-related diseases (Garg and Gupta, 2016).

Green coffee beans, known for their potential health benefits, have attracted attention from the cosmetic oil industry primarily due to the positive skin effects associated with their oil, such as hydration and protection from solar radiation (Hussein and Ramadan, 2020). Green coffee oil can be extracted by various methods such as mechanical pressing (Turatti, 2001), supercritical CO_2_ extraction (De Oliveira et al., 2014), microwave-assisted extraction (Tsukui et al., 2014), and more recently, by using aqueous (AEP) and enzyme-assisted aqueous extraction processes (EAEP) (Souza Almeida et al., 2021). In addition to oil recovery, AEP and EAEP enable the co-extraction of other compounds such as proteins and their hydrolysates, carbohydrates, antioxidants, and methylxanthines into the reaction medium. By enabling the concurrent extraction of multiple compounds from green coffee beans, these eco-friendly strategies can be tailored to expand and unlock novel industrial applications from this natural and rich resource.

Interestingly, using enzymes during the extraction process can release protein fragments and bioactive peptides, which can significantly impact the biological properties of the extracts (Görgüç et al., 2020). For example, flaxseed protein hydrolysates generated using proteases such as Alcalase, Neutrase, and Protamex have shown stronger antioxidant potential and mild antiproliferative activity compared with when not using enzymes, especially when Alcalase protease was employed in the process (Logarušić et al., 2020). Moreover, peptides derived from peanut (Jamdar et al., 2010), quinoa (Mudgil et al., 2020), and flaxseed proteins (Marambe et al., 2008) are examples of compounds capable of inhibiting the angiotensin-converting enzyme (ACE), therefore, presenting anti-hypertension properties. These peptides, which typically contain from two to nine amino acids, are usually rich in hydrophobic amino acid residues and often feature proline, lysine, or arginine as the C-terminal amino acid (Kitts and Weiler, 2003; Bessada et al., 2019).

The consumption of green coffee extracts has been associated with desirable antioxidant activity and the inhibition of mutagenicity of carcinogens. Additionally, green coffee extracts have shown effects on body mass, blood glucose, lipid levels, and blood pressure (Sanlier et al., 2019). The antidiabetic properties of green coffee bean extracts have been attributed to the presence of chlorogenic acids, which play an important role in inhibiting α-glucosidase activity, therefore slowing down the absorption of glucose (Garg and Gupta, 2016; Sanlier et al., 2019). The anti-obesity activity of green coffee bean extracts has been related to the presence of both caffeine and chlorogenic acids. A previous study has demonstrated that mice fed with green coffee extract had a positive effect against gain of body weight and fat accumulation due to the role of caffeine as a fat absorption suppressor and that of chlorogenic acid and its related compounds in enhancing fat metabolism in the liver (Shimoda et al., 2006). Although the antihypertensive effect of green coffee bean extracts has been mainly associated with the presence of chlorogenic acids (Watanabe et al., 2006; Suzuki et al., 2002), it has recently been reported that the 11S coffee globulin is a precursor of a series of bioactive peptides that lead to anti-hypertensive and antioxidant activities (Ribeiro et al., 2021). Thus, careful consideration should be given to the impact of protein extraction (aqueous vs. enzymatic), recovery methods, and purification steps, as they can alter the composition, concentration, and therefore, the bioactivity of green coffee bean peptides (Daliri and Lee, 2017), which could be explored as valuable nutraceutical compounds (Souza Almeida et al., 2021; Almeida et al., 2022).

The biological properties of selected extracts are influenced by the extraction methods and conditions employed (Yan et al., 2022). Our research team has recently elucidated how process conditions affect protein and lipid extractability (Souza Almeida et al., 2021) and protein techno-functionality (Almeida et al., 2022). We developed processing strategies that resulted in 80% reduction in enzyme usage and 60% in water usage. These processes yielded proteins with unique properties, including low molecular weight, high solubility and good emulsifying properties (Almeida et al., 2022). However, the impact of extraction methods and conditions on the biological properties of coffee extracts has yet to be investigated. Therefore, we hypothesized that enzymatic extractions could not only enhance protein and lipid extractability and protein functionality, but also release other compounds, such as phenolics. Furthermore, proteolysis can lead to molecular changes during extraction, including the release of peptides, ultimate resulting in extracts with high biological activity.

In this context, the overall goal of this study was to evaluate the impact of the aqueous (AEP) and enzyme-assisted aqueous extraction processes (EAEP) on the biological and nutritional properties of the extracts (protein- and antioxidant-rich fraction) produced from Arabica green coffee beans. The role of extraction pH in the AEP and various enzymatic strategies in the EAEP were evaluated with respect to their impact on *in vitro* protein digestibility, antioxidant, and antimicrobial activity, as well as the inhibitory effects of the extracts against α-glucosidase, pancreatic lipase, and angiotensin-converting enzyme. The elucidation of the impact of sustainable extraction approaches such as AEP and EAEP, which can simultaneously extract lipids, proteins, carbohydrates, and antioxidant phenolics from green coffee beans without the use of flammable solvents, is of paramount importance for the development of bioprocessing strategies to produce green coffee extracts with tailored biological and nutritional properties.

## 2 Material and methods

### 2.1 Material

Arabica green coffee beans were acquired from the Brazilian Cerrado (#Lot 10/511-16, Genuine Origin, USA). The enzymes Neutral Protease 2 million (NP) (#252-10134-1-RB), Cellulase (C) (#255-10014-1-RB), and Hemicellulase (H) (#T76135-50 mg-TM) were kindly donated by Biocat (Troy, VA, USA), while alkaline protease was donated by Danisco (#216661-9.13 EN, Danisco, Rochester, USA). Amylase (#86250), pepsin (#10108057001), pancreatin (#P1625), bile salts (#B8756), lipase from porcine pancreas Type II (#L3126), Folin-Ciocalteu reagent (#F9252), Trolox (#238813), 2,2′-Azino-bis (3-ethylbenzothiazoline-6-sulphonic acid) (ABTS) (#A1888), potassium persulfate (#906735), fluorescein (#46955), 2,2′-Azobis (2 methylpropionamidine) dihydrochloride (AAPH) (#440914), α-glucosidase (#G0660), p-nitrophenyl laurate (pNP laurate) (#61716), p-Nitrophenyl α-D-Glucoside (p-NPG) (#S965839), Triton X-100 (#1.08603), angiotensin-converting enzyme (ACE) from rabbit lung (#A6778), Captopril (#1091200), Acarbose (#A8980), phosphoric acid (≥85%) (#695017), hydrochloric acid (≥36%) (#258148), methanol (HPLC grade) (#646377), acetonitrile (HPLC grade) (34998, Sigma-Aldrich, St. Louis, MO, USA), gallic acid (≥99%) (#27645), chlorogenic acid (≥95%) (#00500590), quercetin (≥95%) (#PHL89262), caffeic acid (≥98%) (#C0625), (−) epicatechin (≥98%) (#E4018), ferulic acid (≥95%) (#1270311), vanillin (≥97%) (#W310700), m-coumaric acid (≥99%) (#92649), trans-ferulic acid (≥99%) (#W518301), o-coumaric acid (≥97%) (#PHL82343), caffeine (≥99%) (#W222402), resveratrol (≥99%) (#R5010), and p-coumaric acid (≥98%) (#03200595) were acquired from Sigma-Aldrich (St. Louis, MO, USA). The reagent o‐aminobenzoylglycyl‐p‐nitro‐L‐phenylalanyl‐L‐proline was purchased from Peptides International (#FA65740, New England Peptide, Inc., Gardener, MA, USA). Gallic acid for total phenolic compounds evaluation was acquired from Chem Impex International Inc (#22784, Chem Impex International Inc, Bensenville, IL, USA). The culture media Trypticase Soy Agar (TSA) and Tryptic Soy Broth (TSB) were from BD BBL (#B12305, #DF0370-17-3, Thermo Fisher Scientific, Waltham, WA, USA). The bacterial strains *Staphylococcus aureus* (ATCC 51740), *Salmonella typhimurium* (ATCC 29630), *Enterococcus faecalis* (ATCC, 19433), *Bacillus cereus* (ATCC 11778), *Listeria monocytogenes* (ATCC, 19115) and *Escherichia coli* (ATCC 10798) were obtained from the American Type Culture Collection (Manassas, VA, USA). All the other chemicals used were of analytical grade.

### 2.2 Production of green coffee extracts

Coffee extracts were obtained by either aqueous (AEP) or enzyme-assisted aqueous extraction processes (EAEP) as described in our previous study (Souza Almeida et al., 2021). Briefly, aqueous extractions were performed at 1:10 (w/v) solids-to-liquids ratio (SLR), under constant stirring of 120 rpm at 50 °C for 60 min, either at pH 7.0 or 9.0. For the EAEP, alkaline protease (AP), neutral protease (NP), cellulase (C), and hemicellulase (H) were used alone or in combination. Therefore, the EAEP slurry pH was selected based on the optimal pH of the enzyme used, as suggested by the manufacturer's recommendations. AEP and EAEP extracts were generated according to the conditions described in Table 1.

**Table 1:** Extraction conditions for producing AEP and EAEP green coffee extracts.

Experiment	%Enzyme (w/w)^a^	pH	Time (min)	SLR (w/v)	Temperature (°C)
NP-60 min	0.5% NP	7.0	60	1:10	50
AP-60 min	0.5% AP	9.0	60	1:10	50
NP-30 min	0.5% NP	7.0	30	1:10	50
AP-30 min	0.5% AP	9.0	30	1:10	50
(C + H)^b^ + NP	0.25% C + 0.25% H	5.6	30	1:10	50
	0.5% NP	7.0	30		
(C + H)^b^ + AP	0.25% C + 0.25% H	5.6	30	1:10	50
	0.5% AP	9.0	30		
AEP – pH 7.0	–	7.0	60	1:10	50
AEP – pH 9.0	–	9.0	60	1:10	50

a (weight of enzyme/weight of flour).

b Pretreatment with carbohydrases followed by protease extraction.

After extraction, the slurry was centrifuged in a centrifuge (Allegra X-14 R, Beckman Coulter, Brea, USA) at 4000×*g* for 30 min at 4 °C to separate the insoluble fraction from the liquid phase (cream – oil-rich phase and aqueous phase). Subsequently, the cream layer was separated from the aqueous phase using a separatory funnel under refrigerated conditions overnight, resulting in the generation of the extracts for further analyses. Only the AEP and EAEP aqueous extracts (protein and antioxidant-rich aqueous phase) were evaluated in this study. They were kept frozen at −20 °C until nutritional and biological characterization. Three independent batches were produced (n = 3) for biological properties characterization.

### *2.3* *In vitro* protein digestibility

The *in vitro* protein digestibility of the extracts was investigated as previously described (de Souza et al., 2020; Bornhorst and Singh, 2013). Briefly, the oral condition was simulated by mixing 3.33 mL of Simulated Saliva Fluid (SSF) (Table 1S) with 5 mL of liquid skim fractions. Then, 6.67 mL of Simulated Gastric Fluid (SGF) (Table 1S) was added to the previous mixture, followed by pH adjustment to 3.0. Samples were incubated into a water-bath under stirring at 140 rpm for 2 h at 37 °C. Finally, the intestinal condition consisted of adding Simulated Intestinal Fluid (SIF) (Table 1S) to a 1:1 (v/v) ratio. The pH was adjusted to 7.0 and samples were next incubated for 2h, under the same described conditions. The protein content of the undigested samples and that of the pellet left after digestion were measured and used to calculate the amount of protein in the sample before and after digestion, as described in Eq. (1). To recover the pellet after digestion, the digested protein was precipitated (1:1 v/v) with a 24 % (w/v) trichloroacetic acid solution and centrifuged at 4000 rpm for 30 min at 4 °C in a centrifuge (Allegra X-14 R, Beckman Coulter, Brea, USA). The precipitated proteins were quantified using a Nitrogen Analyzer (N × 5.24) (Vario MAX cube, Elementar Analysensysteme GmbH, Langenselbold, Germany), in duplicate.

(Eq. 1) $$Digestibility\;\left(\%\right)=100\;x\;\left(\;\frac{{Protein}_{extract}-\left({Protein\;}_{pellet}-{Protein\;}_{control}\right)}{{Protein}_{extract}}\right)$$

In addition, 500 μL aliquots were taken at the end of each digestion phase to evaluate the molecular weight profile of proteins/peptides by sodium dodecyl sulphate-polyacrylamide gel electrophoresis (SDS-PAGE) using Criterion TM TGX Precast Gels (Bio Rad, Hercules, CA, USA), as previously described (Souza Almeida et al., 2021). A standard with molecular mass ranging between 10 and 250 kDa (Bio Rad, Hercules, CA, USA) was used and the distribution of proteins and peptides was then analyzed by a Gel Imager and Image Lab software (Gel DOCTM EZ, Bio-Rad, Hercules, USA). The digestibility assays were performed in duplicates.

### 2.4 Total phenolic content (TPC)

The total phenolic content of the extracts was determined according to the Folin-Ciocalteau spectrophotometric method, as previously described (Singleton et al., 1999). Briefly, an aliquot of 25 μL of the extract solution (1:50 v/v, extract:water, ∼0.75 mg of solids/mL) and 125 μL of the Folin-Ciocalteau aqueous solution (1:10 v/v) were transferred to a microplate well. The mixture was stirred at 300 rpm for 5 min at room temperature. Then, 100 μL of a 7.5% (w/v) sodium carbonate solution was added to the mixture, which was subsequently stirred at 300 rpm for 30 min and kept in the dark at room temperature for 90 min (totaling 2 h of reaction time). The absorbance was read at 760 nm using a spectrophotometer (SpectraMax iD5, Molecular Devices, San Jose, USA). The results were calculated from the gallic acid standard curve (R^2^ = 0.9995) with concentrations ranging from 5 to 80 μg/mL and expressed as mg of gallic acid equivalent (GAE)/100 g dried extract. Each extraction replicate (n = 3) was analyzed in triplicate.

### 2.5 Phenolic profile of AEP and EAEP extracts by high-performance liquid chromatography coupled with diode array detection (HPLC-DAD)

Prior to analysis, extracts were weighed (10 mg ± 0.001) and diluted in a solution of 1:1 methanol: water + 0.1% HCl. The solutions were sonicated in an ultrasonic bath (404, DA, Delta Ultrassons, Diadema, Brazil) until complete dissolution. After that, a centrifugation step was performed in a benchtop centrifuge (Excelsa i2206, Fanem, Guarulhos, Brazil) at 2202×*g*. The supernatant was collected and filtered through 20 μm membranes, and then the samples were injected into an HPLC system.

For the separation and quantification of analytes, a previous method was employed with some modifications (Pinton et al., 2022). A high-performance liquid chromatography system (LC20AD, Shimadzu, Kyoto, Japan), equipped with a Shimadzu autoinjector and detector (SIL20AHT, SPD-6AV, Shimadzu, Kyoto, Japan), and an Agilent Eclipse column (4.6 mm × 250 mm with 5 μm particle size) (XDB-C18, Agilent, Santa Clara, USA) was used at 35 °C. The mobile phases used were: mobile phase A, composed of MilliQ water and 1.0% (v/v) phosphoric acid, and mobile phase B, composed of 80% (v/v) acetonitrile and 20% (v/v) mobile phase A. The concentration gradient involved increasing mobile phase B from 10 to 31% (0–73 min) and then to 62% (73–75 min). Afterward, mobile phase B was kept at 62% until 80 min and then reduced to the initial 10% from 82 to 90 min. The flow rate of the mobile phase in the system was 1 mL/min, and the sample injection volume was 20 μL. The eluted compounds were monitored and identified by comparing their UV–vis spectra and retention times with standards: gallic acid (214 nm), chlorogenic acid (204 nm), quercetin (325 nm), caffeic acid (323 nm), (−)epicatechin (220 nm), vanillin (230 nm), m-cumaric acid (309 nm), trans-ferrulic acid (322 nm), o-cumaric acid (277 nm), caffeine (278 nm), resveratrol (305 nm), p-cumaric acid (370 nm). The calibration curves were prepared in a 1:1 methanol:acidified water within the 3–73 mg/mL concentration range for all standards. The curve slope, intercept and fit can be found in the Supplementary Material (Table 2S). Each extract (n = 3) was analyzed in triplicate.

**Table 2:** Total polyphenol content, antioxidant activity, and digestibility of AEP and EAEP green coffee extracts.

Sample	TPC (mg gallic acid equivalent (GAE)/100 g dry extract)	TEAC (mmol Trolox equivalent/g dry extract)	FRAP (mmol Trolox equivalent/g dry extract)	ORAC (mmol Trolox equivalent/g dry extract)	Protein content (%) dry basis	Protein digestibility∗ (%)
NP-60 min	9730.0 ± 344.7^a,b^	35.4 ± 0.3^a,b^	609.5 ± 2.2^a,b^	1.18 ± 0.07^a^	25.90 ± 1.48^a^	99.03 ± 0.41^a^
AP-60 min	7694.0 ± 191.3^c,d^	26.0 ± 0.8^c^	346.2 ± 8.6^c,d^	0.89 ± 0.10^b^	24.85 ± 0.44^a,b^	99.36 ± 0.47^a^
NP-30 min	8757.0 ± 498.8^a,d^	34.8 ± 0.3^a,b^	593.3 ± 50.7^a,b,c^	1.17 ± 0.09^a^	24.80 ± 0.56^a,b^	99.88 ± 0.04^a^
AP-30 min	8517.0 ± 137.8^b,d^	28.3 ± 0.3^b,c^	387.1 ± 29.2^b,d^	0.83 ± 0.10^b^	24.68 ± 0.17^a,b^	100.12 ± 0.68^a^
(C + H) + NP	8861.0 ± 65.1^a,b,c^	33.2 ± 0.2^a,c^	598.4 ± 10.2^a,b^	0.82 ± 0.07^b^	22.88 ± 0.51^b^	99.48 ± 0.12^a^
(C + H) + AP	8243.7 ± 129.5^c,d^	25.7 ± 0.2^c^	396.9 ± 15.9^b,d^	0.50 ± 0.05^c,d^	22.84 ± 0.47^b^	99.94 ± 0.35^a^
AEP-pH 7.0–60 min	10160.3 ± 358.5^a^	38.5 ± 0.2^a^	690.6 ± 20.3^a^	0.61 ± 0.06^c^	23.95 ± 1.01^a,b^	98.83 ± 0.14^a^
AEP-pH 9.0–60 min	7666.0 ± 318.2^d^	27.6 ± 0.8^b,c^	326.5 ± 15.6^d^	0.46 ± 0.12^d^	23.61 ± 0.74^b^	100.79 ± 0.64^a^

Different letters within the same column indicate significant differences among samples (p < 0.05).∗ *in vitro*

### 2.6 Antioxidant activity of AEP and EAEP coffee extracts

#### 2.6.1 Ferric reducing antioxidant power (FRAP) assay

The ferric reducing antioxidant power (FRAP) assay was performed according to the previous methodology (Benzie and Strain, 1996). The FRAP reagent was prepared by mixing 25 mL of 0.3 M acetate buffer (pH 3.6) with 2.5 mL 2,4,6-Tris(2-piridil)-s-triazina (TPTZ, 10 mM in 40 mM HCl) and 2.5 mL FeCl_3_ (20 mM) solutions. Twenty-microliter aliquots of diluted extracts (1:50 v/v, extract/water, ∼0.75 mg of solids/mL) were transferred to a 96-well microplate containing 150 μL of FRAP reagent. The mixture was then stirred and kept at rest for 10 min in the dark at room temperature. Absorbance was read at 593 nm using a spectrophotometer (SpectraMax iD5, Molecular Devices, San Jose, USA). A standard calibration curve (y = 0.0021x + 0.0085, R^2^ = 0.9997) using Trolox (15–600 μmol/mL) was used for quantification purposes, and the results were expressed as mmol Trolox equivalent/g dried extract. All AEP and EAEP extracts (n = 3) were analyzed in triplicate.

#### 2.6.2 Trolox equivalent antioxidant capacity (TEAC) assay

The TEAC assay also known as the ABTS•^+^ radical cation scavenging activity was carried out according to the procedure previously reported by Al-Duais et al. (2009) with some modifications. The ABTS•^+^ stock solution (radical) was initially produced by mixing a 3.84 mg/mL ABTS solution with 0.662 mg/mL K_2_S_2_O_8_. The radical was incubated for 24 h at room temperature in the dark to reach the optimum concentration in the mixture. Then, the stock radical solution was diluted with ethanol to obtain an initial absorbance of 0.70 ± 0.20 at 730 nm. The analysis was performed by pipetting 20 μL of each 1:5 (v/v extract:water, ∼7.5 mg of solids/mL) diluted sample into a microplate containing 200 μL of fresh diluted ABTS•^+^ solution. A control with deionized water was also prepared. The mixtures were stirred and kept at rest for 6 min in the dark at room temperature for further absorbance readings at 730 nm using a spectrophotometer (SpectraMax iD5, Molecular Devices, San Jose, USA). A calibration curve (y = −0.1009x + 0.6084, R^2^ = 0.998) was constructed using Trolox solutions (80–300 μM) prepared in ethanol and the results were expressed as mmol Trolox equivalent/g dried extract. All AEP and EAEP extracts (n = 3) were analyzed in triplicate.

#### 2.6.3 Oxygen radical absorbance capacity (ORAC) assay

The ORAC assay was performed according to the procedure described by Zulueta et al. (2009). Briefly, 50 μL of fluorescein (78 nM) and 50 μL of diluted extract (1:5000 v/v extract:water, ∼0.0075 mg of solids/mL) in 0.1 M phosphate-buffered saline solution (PBS) at pH 7.0, along with blank (PBS) or standard (Trolox, 20 μM in PBS), were placed in a 96-well microplate. Subsequently, 25 μL of 221 mM AAPH solution (radical) was added. A microplate reader (SpectraMax iD5, Molecular Devices, San Jose, USA) was used for the readings at excitation wavelength of 485 nm and emission wavelength of 535 nm. The fluorescence measurements were then taken every 5 min, at 37 °C, until the relative fluorescence intensity was less than 5% of the initial value. All AEP and EAEP extracts (n = 3) were analyzed in triplicate. The ORAC values were expressed as mmol Trolox equivalent (TE)/g dried extract and were calculated as follows (Eq. (2)):

(Eq. 2) $$ORAC\;\left(\mu M\;TE\right)=\frac{C_{Trolox}*\left({AUC}_{Sample}-{AUC}_{Blank}\right)*DF}{\left({AUC}_{Trolox}-{AUC}_{Blank}\right)}$$

where C_Trolox_ represents the concentration of the Trolox standard (20 μM), DF is the sample dilution factor (5000), and AUC is the area under the fluorescence decay curve of the sample, blank, and Trolox, respectively. Samples were analyzed in triplicate.

### 2.7 Inhibitory potential of AEP and EAEP green coffee extracts against enzymes

#### 2.7.1 Angiotensin-converting-enzyme (ACE) inhibitory activity

The ACE inhibitory activity of the different extracts was measured according to a method previously developed (Sentandreu and Toldrá, 2006), which is based on the ability of ACE to hydrolyze the internally quenched fluorescent substrate o‐aminobenzoylglycyl‐p‐nitro‐L‐phenylalanyl‐L‐proline (Abz‐Gly‐Phe‐(NO2)‐Pro). For this, a 50 μL aliquot of the extract, diluted 1:30 (v/v) (∼1.25 mg of solids/mL) in a 150 mM Tris buffer (pH 8.3), was mixed with 50 μL of ACE enzyme solution containing 30 μm/mL of ACE, also dissolved in 150 mM Tris buffer (pH 8.3). The mixture was pre-incubated for 10 min at 37 °C. The substrate was pre-incubated apart at the same temperature. The reaction was initiated by adding 200 μL of 10 mM Abz‐Gly‐Phe‐(NO_2_)‐Pro in a 0.15 M Tris‐base buffer (pH 8.3) containing 1.125 M NaCl. The mixture was then incubated at 37 °C for 30 min. The amount of aminobenzoylglycine (Abz‐Gly) formed by ACE activity was measured using a microplate reader (SpectraMax iD5, Molecular Devices, San Jose, USA) at 355 and 428 nm as excitation and emission wavelengths, respectively. ACE inhibitory activities of the samples were expressed as a decrease in fluorescence compared to the negative control – extract replaced by 150 mM Tris-base buffer (pH 8.3) (Eq. (3)). For comparison, Captopril 0.08 μM (1.7 × 10^−5^ mg/mL) was used as a positive control.

(Eq. 3) $$Inhibitory\;activity\;\left(\%\right)=100\times \left[1-\frac{{Fluo}_{S}}{{Fluo}_{C}}\;\right]$$

where Fluo_C_ corresponds to the fluorescence measurement for the control (enzyme + substrate + Tris-base buffer); Fluo_S_ is the fluorescence for the sample (extract + substrate + enzyme). Each process replicate (n = 3) was analyzed in triplicate (n = 3).

#### 2.7.2 Lipase activity inhibition assay

The lipase inhibitory activity assay was performed as previously described by McDougall and collaborators (McDougall et al., 2009). Initially, a 10 mg/mL enzyme solution was prepared by dissolving the lipase in ultra-pure water. The solution was next centrifuged at 1500×*g* for 10 min at 20 °C and the supernatant was recovered for subsequent use. The substrate consisted of 0.08% (w/v) p-nitrophenyl laurate (pNP laurate) dissolved into 5 mM sodium acetate (pH 5.0) containing 1% Triton X-100. This solution was heated in boiling water for 1 min to facilitate dissolution. The assay consisted of mixing 400 μL pre-diluted samples (1:5 extract/water, ∼7.5 mg of solids/mL) or buffer assay (100 mM Tris buffer, pH 8.2, control reaction) with 450 μL substrate solution and 150 μL lipase. The reaction was carried out for 2 h, at 37 °C. Samples were next centrifuged at 13,226×*g* for 3 min and the supernatant was analyzed at 400 nm in a microplate reader (SpectraMax iD5, Molecular Devices, San Jose, USA). All AEP and EAEP extracts (n = 3) were analyzed in triplicate and a sample background blank was prepared for each sample by replacing the enzyme with water. The pancreatic lipase inhibitory activity was calculated as described by Eq (4).

(Eq. 4) $$Inhibitory\;activity\;\left(\%\right)=100\times \left[1\;˗\;\frac{{ABS}_{S}-{ABS}_{SB}}{{ABS}_{C}}\;\right]$$

where ABS_s_ corresponds to the sample reaction (enzyme + substrate + extract); ABS_sb_ is the sample background (extract + substrate, without enzyme), and AB_Sc_ is the control reaction (no extract addition: Enzyme + substrate + water). Each process replicate (n = 3) was analyzed in triplicate (n = 3).

#### 2.7.3 α -glucosidase inhibitory activity

The α-glucosidase inhibitory activity was evaluated according to the methodology described Ibrahim et al. (2018), with modifications. Initially, 50 μL of each extract was diluted in water (1:5 v/v) to achieve a final concentration of 0.75 mg of solids/mL. Acarbose was also diluted and used as a positive control. Then, 25 μL of the dilutions were incubated with 0.5 U·mL^−1^ α-glucosidase solution from *Saccharomyces cerevisiae,* in phosphate buffer (PBS, 100 mM, pH 6.8) at 37 °C, for 5 min.

After pre-incubation, 25 μL of p-nitrophenyl-α-d-glucopyranoside (p-NPG) substrate solution (5 mM) in PBS (100 mM, pH 6.8) was added, and the reaction was allowed to proceed for 10 min, at 37 °C. The absorbance was measured at 405 nm in a microplate reader using a spectrophotometer (SpectraMax iD5, Molecular Devices, San Jose, USA). A control reaction and a sample background were used to calculate the inhibitory activity as described in Eq. (4) and reported previously by Kee et al. (2013). Each process replicate (n = 3) was analyzed in triplicate (n = 3).

### 2.8 Antimicrobial activity

#### 2.8.1 Screening

For the antimicrobial screening assay, the Gram-positive bacterial strains *S. aureus*, *B. cereus*, *E. faecalis,* and *L. monocytogenes* and the Gram-negative bacterial strains *E. coli* and *S. typhimurim* were revived in Tryptic Soy Broth (TSB) and consecutively sub-cultured with 24 h intervals on Trypticase Soy Agar (TSA) plates at 37 °C. After the activation, TSB cultures were grown until 0.5 McFarland standard. A 2.5 mL aliquot of each culture was mixed with either 2.5 mL of extract or 2.5 mL of sterile water (control) and incubated overnight at 37 °C, under agitation. Following incubation, each mixture was serially diluted and 10 μL aliquots were spotted onto square TSA plates with grids, which were then incubated overnight and observed. Strains showing visible reduction in growth compared to controls were used in subsequent quantitative assays.

#### 2.8.2 Quantitative assay

For the antimicrobial quantitative assay, *E. coli* and *S. aureus* cultures were grown in TSB at 37 °C until 0.5 McFarland standard. Next, 2.5 mL of each culture was mixed with either 2.5 mL of extract or 2.5 mL of sterile water (control) and incubated overnight at 37 °C, under agitation. Following incubation, each mixture was serially diluted, and 0.1 mL of dilutions 10^−5^ and 10^−6^ were spread onto TSA plates in triplicate. Plates were incubated overnight at 37 °C and CFU·mL^−1^ were counted. The plates containing the extracts were compared against control plates to calculate the surviving fraction.

### 2.9 Statistical analysis

Statistical analysis was performed using the GraphPad Prism software (version 10.3.1, GraphPad Software Inc., Boston, MA, USA). Samples that followed a normal distribution with homoscedastic variance were analyzed using Two-way ANOVA, followed by Tukey's post hoc test to assess differences between the means. For non-normal data with heterogeneous variance, non-parametric tests, including the Kruskal-Wallis test and the Dunn-Bonferroni post hoc test, were applied. All statistical tests had the significance level set at p < 0.05.

## 3 Results and discussion

### *3.1* *In vitro* digestibility of AEP and EAEP coffee proteins

**Fig. 1:**
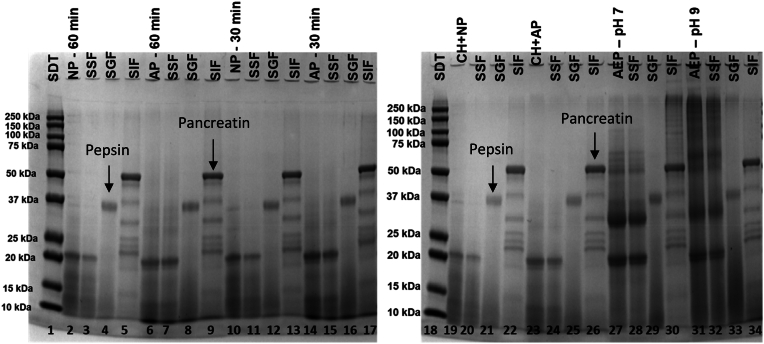
*In vitro* protein digestibility of coffee extracts evaluated by SDS-PAGE gel: lane 1 and 18 represent the protein molecular weight standards; lane 2, 6, 10, 14, 19, 23, 27 and 31 represent the extracts before digestion for the AEP extracts at pH 7.0 and 9.0 and EAEP extracts (NP – Neutral protease; AP – Alkaline protease, and CH – carbohydrases). SSF, SGF, and SIF represent samples collected after simulated oral, gastric, and intestinal digestion, respectively.

The impact of different enzymatic treatments on the electrophoretic distribution and digestibility of coffee proteins was assessed (Fig. 1). According to the SDS-PAGE gel, AEP extracts (without enzyme) obtained at neutral (AEP – pH 7.0, lane 27) and alkaline pH (AEP – pH 9.0, lane 31) presented two main bands at 19.5 and 31 kDa before digestion, which can be attributed to the 11S storage protein fractions (α and β coffee legumins), under denaturing conditions (Acuña et al., 1999). These proteins account to 45% of the total protein in the coffee endosperm (Rogers et al., 1999). Moreover, bands between 50 and 250 kDa were also observed in the SDS-PAGE gels for AEP extracts (Fig. 1, lanes 27 and 31); however, they were no longer observed when either neutral (NP) or alkaline (AP) proteases were used during extractions (Fig. 1, lanes 2, 6, 10, 14, 19, and 23), indicating that they were completely degraded along with the β-legumin band (31 kDa).

Despite the high polyphenol content of the extracts (as shown in Table 2, Table 3), high *in vitro* protein digestibility values were observed after the intestinal phase (average ∼99%). Therefore, the potential inhibitory effects of polyphenols on protein digestibility (Cirkovic Velickovic and Stanic-Vucinic, 2018) did not significantly alter the digestibility of green coffee proteins, nor did the different extraction conditions employed (Table 2). The high digestibility of the proteins in the AEP and EAEP extracts is likely related to the low molecular weight of coffee proteins, as smaller proteins and peptides facilitate the access of digestive enzymes (e.g. pepsin and pancreatin) to the peptide bonds.

**Table 3:** Phenolic and caffeine content in the extracts (mg/100 g dry extract).

Sample	NP-60 min	AP-60 min	NP-30 min	AP-30 min	(C + H) + NP	(C + H) + AP	AEP pH 7	AEP pH 9
**Gallic Acid**	118.08 ± 5.84^a^	102.1 ± 8.84^a^	121.02 ± 0.83^a^	120.58 ± 2.19^a^	111.03 ± 9.95^a^	115.45 ± 8.59^a^	115.45 ± 8.59^a^	102.95 ± 4.95^a^
**Chlorogenic acid**	6194.13 ± 86.47^a,b^	4726.27 ± 138.35^d^	5987.01 ± 8.54^b^	5394.6 ± 122.95c	6030.21 ± 53.96^a,b^	5198.8 ± 150.75^c^	6335.32 ± 142.02^a^	4323.29 ± 120.64^e^
**Quercetin**	<LOD	<LOD	<LOD	<LOD	<LOD	<LOD	<LOD	<LOD
**Caffeic acid**	130.88 ± 1.71^a,b^	90.24 ± 3.21^b^	136.53 ± 1.53^a,b^	117.22 ± 7.23 ^a,b^	153.85 ± 10.14^a^	99.27 ± 3.53^a,b^	129.54 ± 4.04^a,b^	85.73 ± 3.05^b^
**Epicatechin**	1208.12 ± 49.05^c^	1234.76 ± 22.89^b,c^	1011.32 ± 9.6^d^	862.8 ± 59.71^e^	1042.89 ± 38.7^d^	1364.63 ± 14.51^a^	1325.41 ± 34.21^a,b^	1173.03 ± 21.75^c^
**Cinnamic acid**	32.46 ± 1.85^a^	23.74 ± 1.35^b^	31.1 ± 1.77^a^	31.4 ± 1.14^a^	35.05 ± 1.23^a^	33.96 ± 1.47^a^	32.8 ± 1.03^a^	25.36 ± 1.37^b^
**Vanillin**	N.D.	N.D.	N.D.	N.D.	N.D.	N.D.	N.D.	N.D.
**m-Cumaric acid**	<LOD	<LOD	<LOD	<LOD	<LOD	<LOD	<LOD	<LOD
**Ferulic acid**	329.34 ± 12.49^a^	126.54 ± 7.36^b^	263.74 ± 17.3^a,b^	233.77 ± 15.6^a,b^	227.09 ± 19.26^a,b^	139.19 ± 8.1^a,b^	231.44 ± 16.76^a,b^	120.21 ± 6.97^b^
**o-Cumaric acid**	<LOD	<LOD	<LOD	<LOD	<LOD	<LOD	<LOD	<LOD
**Caffeine**	1194.48 ± 83.55^b^	1103.86 ± 115.93^b,c^	1157.83 ± 8.09^b^	1187.82 ± 115.09^b^	1127.21 ± 50.21^b^	1453.36 ± 66.65^a^	1260.22 ± 37.97^a,b^	875.91 ± 107.31^c^
**Resveratrol**	<LOD	<LOD	<LOD	<LOD	<LOD	<LOD	<LOD	<LOD
**Catechin**	631.04 ± 22.48^a,b^	436.9 ± 14.83^a,b^	529.14 ± 13.3^a,b^	462.2 ± 2.51^a,b^	718.28 ± 14.92^a^	480.52 ± 16.35^a,b^	775.22 ± 44.13^a^	415.05 ± 14.09^b^
**p-Cumaric acid**	<LOD	<LOD	<LOD	<LOD	<LOD	<LOD	<LOD	<LOD
**∑ Phenolics**	8611.58 ± 169.94^a,b^	6716.81 ± 133.78^a,b^	8048.75 ± 17.95^a,b^	7191.17 ± 47.45^a,b^	8283.35 ± 88.34^a,b^	7397.86 ± 145.04^a,b^	8912.38 ± 138.8^a^	6220.26 ± 139.68^b^

Lowercase letter within the same line indicate statistical differences (p < 0.05). N.D. = non detected; <LOD = value under the detection limit.

The simulated salivary fluid (SSF) did not promote changes in the protein profile for any samples (Fig. 1). SSF only contains mucin, salts, and α-amylase in its composition (Table 1S, supplementary data), therefore, this step shall not lead to proteolysis. Conversely, after the addition of the simulated gastric fluid (SGF), all bands were highly hydrolyzed regardless of the time, pH, and enzyme used during the extractions. This indicates the high effectiveness of pepsin in hydrolyzing coffee proteins (Table 1S, supplementary data). Finally, although some bands are present in the intestinal fluid digesta, they correspond to the enzymes added in the simulated intestinal fluid (SIF) solution rather than coffee proteins (Fig. 1), as confirmed by a control gel performed without samples (Fig. 1S, supplementary data).

A previous report has indicated high protein digestibility for green coffee flour (86.17%) (Jakubczyk et al., 2018). The lower value found for the flour compared to the AEP/EAEP coffee extracts (∼99%) (Table 2) is likely because the extracts contained proteins that had been already extracted, compared with the flour (a complex matrix containing proteins and other macronutrients). Proteolysis during the extraction of coffee flour has been shown to release smaller and more digestible proteins, as demonstrated by *in vitro* protein digestibility assays (Almeida et al., 2022).

The superior protein digestibility of green coffee proteins can also be evidenced when compared to other legumin-like concentrated/isolated proteins such as lentils (∼83%, alkaline extraction - pH 9) (Barbana and Boye, 2013), chickpeas (80–87%, alkaline extraction - pH 8.5) and soybeans (71%, alkaline extraction - pH 8) (Wang et al., 2010), almonds (73%, alkaline extraction - pH 9) (de Souza et al., 2020), and pea proteins (93%, alkaline extraction - pH 11) (Monsoor and Yusuf, 2002). The differences observed among the concentrates/isolates are probably due to the different plant sources and processing conditions under which they were obtained (Drulyte and Orlien, 2019).

### 3.2 Total phenolic content, polyphenol concentration, and antioxidant capacity of AEP and EAEP coffee extracts

Green coffee beans contain different antioxidant compounds such as alkaloids (e.g. caffeine, melatonin, and trigonelline), diterpenes (cafestol and kahweol), vitamins (vitamin E and B12), minerals (e.g. selenium, copper, zinc and manganese), peptides, and phenolic compounds, which are divided in hydroxy-cinnamic acids (e.g. chlorogenic, caffeic, ferulic, coumaric, and sinapic acids), isoflavones (e.g. formononetin, daidzein, and genistein) and anthocyanins (del Castillo et al., 2019). Therefore, it is expected that extracts prepared from coffee beans will exhibit high antioxidant activity. The impact of the AEP and EAEP on the concentration of major phenolic compounds and the antioxidant capacity of the extracts was evaluated and is presented in Table 2, Table 3.

Even though flammable solvent extractions usually present the highest yields (2059–13058 mg GAE/100 g dry sample) on the recovery of phenolic compounds (Al-Duais et al., 2009; Resende Oliveira et al., 2019; Zengin et al., 2020), our results (TPC and HPLC data) demonstrate good recovery of phenolics. The TPC values of the AEP and EAEP extracts ranged from 7666 to 10160 mg GAE/100 g dry extract when evaluated by spectrophotometer (Table 2) and from 6220 to 8912 mg/100 g dry extract, when quantified by HPLC (Table 3). It is important to highlight that the quantification of the phenolic compounds by HPLC might be underestimated because only a targeted approach was applied using the available standards.

Lower TPC values for aqueous extracts prepared from spent coffee grounds and coffee silverskin were reported by Zengin et al. (2020) (TPC values varied from 2049 to 5686 mg GAE/100 g dry extract), which could be attributed to not only the different starting materials but also the preparation method (ultrasonic bath for 120 min at 20 °C vs AEP/EAEP at 50 °C for 1h).

An increase in extraction pH from 7.0 to 9.0 resulted in a 24.5% decrease in the phenolic content (TPC) of the coffee extracts obtained without enzymes (AEP) (Table 2). It has been previously shown that phenolic acids such as caffeic, chlorogenic, and gallic acids are not stable at alkaline pH values (Friedman and Jürgens, 2000), which might have contributed to the observed decrease. Indeed, according to the phenolic profile (Table 3), chlorogenic acid decreased from 6335.32 to 4323.29 mg/100 g dry extract (p < 0.05) when the extraction pH increased from 7.0 to 9.0. No changes were observed for gallic acid concentrations and caffeic acid (p > 0.05). Other compounds such as catechin, epicatechin, cinnamic acid, besides caffeine, also exhibited reduced levels when extracted in alkaline medium (Table 3). These results highlight the significant role of alkaline pH in reducing the stability of phenolic compounds.

The addition of enzymes did not affect total phenolic extractability (TPC) compared to the respective controls (AEP – pH 7.0 and AEP – pH 9.0) (p > 0.05) (Table 2, Table 3). However, higher concentration of chlorogenic acid was found for the EAEP extracts at pH 9.0 (5394-4726 vs. 4323 mg/100 g dry extract) (Table 3) as well as for epicatechin ((C + H) + AP), cinnamic acid (AP – 30 min and (C + H) + AP), and caffeine (AP – 30 min and (C + H) + AP), compared to the aqueous extraction at pH 9.0. This could be attributed to enhanced matrix degradation at pH 9.0, which may facilitate protein extraction through proteolysis compared to the control (Souza Almeida et al., 2021; Almeida et al., 2022). The diffusion of proteins can enhance the release of phenolics into the aqueous medium due to the formation of a more porous structure. However, in spite of the small increment observed in the release of numerous phenolics by the use of alkaline protease, namely chlorogenic acid (for all EAEP treatments), epicatechin ((C + H) + AP), cinnamic acid (AP-30 min and (C + H) + AP), ferulic acid (AP-30 min) and catechin ((C + H) + AP), compared to the control (AEP, pH 9.0, 60 min), these values were similar or lower than the control (AEP) at pH 7.0, highlighting the benefits of using neutral pH during the extraction.

Only neutral protease, when applied for 60 min, resulted in extracts with higher TPC values compared to those obtained using alkaline protease (Table 2). This result is supported by the antioxidant activity and phenolic profile. In fact, caffeic acid (130.88 vs. 90.24 mg/100 g dry extract), cinnamic acid (32.46 vs. 23.74 mg/100 g dry extract) and ferulic acid (329.34 vs. 126.54 mg/100 g dry extract) were present in higher concentrations when using NP-60 min compared to AP-60 min. However, despite the higher phenolic extractability for neutral protease at 60 min, the amount obtained was similar to that of the control (AEP – pH 7.0), which suggests that the enzymatic treatment, while enhancing the concentration of specific phenolic compounds, did not substantially improve overall extractability compared to the control.

Although previous studies have reported a beneficial effect of a carbohydrase mixture (Viscozyme® L) on increasing the extraction of polyphenols for green yerba mate (Heemann et al., 2019), our study found that a pre-treatment with carbohydrases (cellulose + hemicellulose), followed by either AP or NP, did not significantly (p > 0.05) affect the TPC of the extracts. This lack of effect could be attributed to the specificity of the selected enzymes, substrate complexity, and the different conditions applied such as pH and extraction time. Despite the absence of changes in the overall phenolic extractability, it is worth mentioning that the use of carbohydrases in combination with proteases increased extraction of some individual phenolic compounds, as indicated in the previous paragraph.

The antioxidant activity of the AEP and EAEP extracts is shown in Table 2. Data showed that for FRAP, TEAC and ORAC, an increase in extraction pH from 7.0 to 9.0 resulted in significant reduction of the antioxidant capacities of the extracts. These findings agree with the overall reduction in polyphenol extractability and TPC values obtained at alkaline pH (Table 2, Table 3, respectively). Those results suggest that phenolic degradation at alkaline pH directly affects the antioxidant properties of green coffee extracts.

The correlation coefficient between TEAC and FRAP was relatively high (r_s_ = 0.8095) and in agreement with TPC and total phenolic concentration. In contrast, there was a low correlation between TEAC and ORAC (r_s_ = 0.4048) and FRAP and ORAC (r_s_ = 0.3095) assays. These differences found between the methods can be explained by the principle of each assay. ABTS or TEAC assay measures the ability of electron transfer capacity of the antioxidant to the ABTS^•+^ radical, while FRAP assess the ability of antioxidants to reduce iron from Fe^3+^ to Fe^2+^ at low pH values. The ORAC test, on the other hand, measures the antioxidant inhibition of peroxyl radical induced oxidations and, consequently, it evaluates the hydrogen atom transfer capacity (Prior et al., 2005).

Most of the potential health benefits attributed to green coffee is often associated with the presence of phenolic compounds that possess antioxidant properties (Resende Oliveira et al., 2019). Nevertheless, a recent study has reported that the reducing capacity of green coffee is also related to other electron-donating compounds such as proteins and peptides (Ribeiro et al., 2021). Their *in silico* investigation pointed out that antioxidant peptide sequences (e.g. IKK; LY; AH; EL; PHW; PHY; RHQ; VKL; KD; YVL; IR; LK; TY; TFE; VY; FLPE; WG; VYV) can be identified from the 11S globular storage protein; however, in a lower frequency of observation than the ones found for other biological activities (Ribeiro et al., 2021).

It is known that intact proteins can also present antioxidant activity, however, in lower intensity when compared with peptides produced through hydrolytic reactions (Elias et al., 2008). Consequently, the high antioxidant capacity observed in the AEP and EAEP green coffee extracts in our work is likely attributed not only to the phenolic compounds but also to the coffee proteins and peptides present in the extracts (Protein content: 22.84–25.90% dry basis with a degree of hydrolysis (DH) ranging from 13.5 to 31.1%). Therefore, a synergistic effect between peptides and non-peptide antioxidants (e.g., phenolic compounds) could potentially enhance the overall antioxidant capacity (Kitts and Weiler, 2003).

### 3.3 ACE inhibitory activity

The angiotensin-converting enzyme (ACE) is considered a key enzyme of the renin-angiotensin system because it inactivates the vasodilator peptide bradykinin and it cleaves angiotensin I into the vasoconstrictor angiotensin II, therefore, increasing blood pressure (Bessada et al., 2019; Sentandreu and Toldrá, 2006). As such, the discovery of compounds capable of inhibiting ACE activity, thereby acting as therapeutic agents against hypertension, holds significant value (Kitts and Weiler, 2003).

**Fig. 2:**
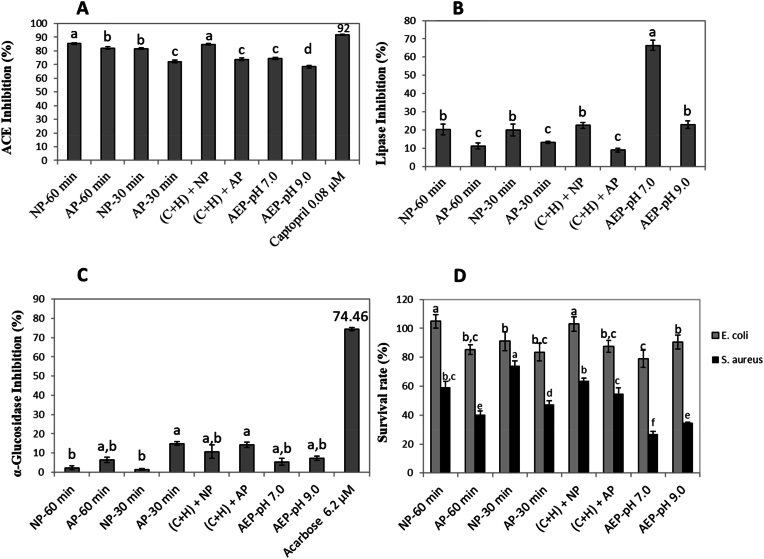
A) Angiotensin-converting enzyme (ACE) inhibitory activity, B) Lipase inhibitory activity, C) α-glucosidase inhibitory activity and D) antimicrobial activity of green coffee extracts obtained through aqueous and enzyme-assisted aqueous extractions. NP-60 min and NP-30 min are the extracts obtained using neutral protease for 60 and 30 min, respectively; AP-30 and AP-60 correspond to the extracts obtained with alkaline protease for 60 and 30 min, respectively; (C + H) + NP and (C + H) + AP are the extracts obtained with a pretreatment containing carbohydrases followed by the addition of either neutral (NP) or alkaline protease (AP); and AEP-pH 7.0 and AEP-pH 9.0 are the controls obtained without the addition of enzymes and at different pH values (pH 7 and 9).

Considering the high concentration of polyphenols in the green coffee extracts (Table 2, Table 3), and the possible presence of coffee protein hydrolysates in the extracts (DH varying from 13.5 to 31.1%) (Souza Almeida et al., 2021), the ACE inhibitory activity of the AEP/EAEP coffee extracts was evaluated (Fig. 2A). All samples, regardless of the extraction method employed, exhibited high ACE inhibitory activity (>85%) (Fig. 2A). However, the extraction methods (AEP vs. EAEP) and conditions (extraction pH and time) significantly influenced the ACE inhibitory activity of the extracts. Moreover, captopril, a synthetic antihypertensive drug, exhibited the highest inhibition rate (92%). Nevertheless, its lower concentration compared to the coffee extracts limits direct comparison with the coffee extracts. Therefore, further studies using IC50 values are recommended for a more accurate assessment.

An increase in extraction pH from 7 to 9, at 60 min of extraction time, decreased ACE inhibitory activity from 74 to 68% (Fig. 2A), agreeing with the reduction observed in TPC values (from 10160 to 7666 mg GAE/100g) and antioxidant capacity (from 38.5 to 27.6 mmol Trolox-Eq/g for TEAC and from 690.6 to 326.5 mmol Trolox-Eq/g for FRAP) (Table 2). Therefore, for the AEP extracts, ACE inhibition seems to be associated mainly with the polyphenol content extracted in each pH condition. In fact, by changing from neutral to alkaline condition, a decrease in chlorogenic acid (6335.3 vs. 4323.3 mg/100g dry extract), epicatechin (1325.4 vs. 1173.0 mg/100 g dry extract), cinnamic acid (32.8 vs. 25.4 mg/100g dry extract), catechin (775.2 vs. 415.0 mg/100 g dry extract) and caffeine (1260.2 vs. 875.9 mg/100 g dry extract) was observed (Table 3). Furthermore, as previously reported (Bhullar et al., 2014), isolated phenolic compounds such as caffeic acid (IC_50_ – 430 μM = 0.0774 mg/mL), quercetin (IC_50_ – 9.85 μM = 0.0029 mg/mL), and chlorogenic acid (IC_50_ – 21.53 μM = 0.0076 mg/mL) present the ability to act as ACE inhibitors. Even though the compounds present in coffee extracts are needed in higher concentrations compared to captopril (IC_50_ = 1.02 μM = 0.00022 mg/mL), their ACE inhibitory activity remains relevant given that they are natural compounds.

The addition of alkaline and neutral proteases to assist the extraction promoted higher inhibitory activity of the extracts compared with their respective controls (AEP-pH 7.0 and AEP-pH 9.0) (Fig. 2A). Even though the protein content of the EAEP extracts was not significantly higher than the AEP treatments (Table 2), the higher DH of the EAEP extracts (Souza Almeida et al., 2021) would likely result in smaller peptides that could potentially inhibit ACE activity. In fact, a recent study demonstrated that when alcalase and thermolysin were employed to hydrolyze a protein extract from coffee beans, peptides with high ACE inhibitory capacity (52.5% and 73.3% inhibition, respectively) were generated (Dai et al., 2023), although lower values were found compared to the present study. Moreover, a previous study on peanut isolates showed ACE inhibition for both the protein isolate (66%) and its hydrolysates (90–97%), with the inhibition capacity increasing with increased hydrolysis (Jamdar et al., 2010). Another study investigated ACE inhibitory activities of quinoa proteins both in their native form and as hydrolysates produced by using the enzymes bromelain, chymotrypsin, and Pronase E. While the non-hydrolyzed proteins presented a very high IC_50_ value of 0.62 mg/mL, the hydrolysates showed IC_50_ values between 0.18 and 0.31 mg/mL, thus indicating strong ACE inhibition for the hydrolysates (Mudgil et al., 2020). Similarly, flaxseed hydrolysates produced by Flavourzyme also exhibited high ACE inhibition. According to the study, the different hydrolysates (11.94–70.62% DH) demonstrated strong ACE inhibition at 0.67 mg/mL concentration (71.59–88.29%), while the non-hydrolyzed proteins presented no ACE inhibitory activity, suggesting that the observed activity was due to the peptides generated from proteolysis (Marambe et al., 2008). Therefore, we hypothesize that not only phenolics but also peptides were effective at inhibiting ACE in the EAEP coffee extracts evaluated in the present study, which highlights the potential of coffee protein hydrolysates to be explored as antihypertensive drugs.

By increasing reaction time from 30 to 60 min in the EAEP, an increase in ACE inhibition was observed when using neutral and alkaline proteases as well as for the pretreatment with cellulases followed by the use of NP (Fig. 2A). Even though the amount of proteins did not differ significantly among samples (22.84–25.90%, dry-basis) (Table 2), the DH of the extracts had a larger variation (13.5–31.1%), where (C + H) + NP presented the highest DH (31.1%), followed by NP 60 min (22.4%) (Souza Almeida et al., 2021). These samples also presented the highest amount of chlorogenic acid in the extracts (Table 3). Consequently, the increase in ACE inhibition might be associated with both the bioactive peptides and phenolic compounds released in the extraction medium during these extractions. The ACE inhibitory activity of peptides relies on its short hydrophobic structure (two to nine amino acids), amino acid sequence, and breakdown by gastrointestinal enzymes (Kitts and Weiler, 2003; Bessada et al., 2019; Ibrahim et al., 2018). In contrast, the ACE inhibitory activity of polyphenols is influenced by the class and structure of the phenolics, number of hydroxyl groups on the benzene ring, and by the substitution of hydroxyl groups with methoxy groups, which can reduce ACE activity (Al Shukor et al., 2013).

Bearing in mind that many synthetic antihypertensive drugs are known to cause side effects (Daliri and Lee, 2017), studies to discover new sources of antihypertensive peptides are of great value, considering they could be exploited as milder and potentially safer replacements for synthetic drugs (Jamdar et al., 2010).

### 3.4 Lipase inhibitory activity

Pancreatic lipase is known for being a key enzyme in lipid digestion, considering it catalyzes the hydrolysis of triacylglycerols into free fatty acids and monoacylglycerols (Narita et al., 2012). Inhibitors of pancreatic lipase such as polyphenols (Buchholz and Melzig, 2015) seem to be effective for preventing obesity because they delay lipolytic reactions and, therefore, control the absorption of fat and the excessive release of free fatty acids that may cause insulin resistance (Jakubczyk et al., 2018; Almoosawi et al., 2010). Thus, AEP and EAEP extracts were evaluated regarding their lipase inhibitory activity (Fig. 2B).

Extraction pH and methods (AEP vs. EAEP) had a significant impact on the lipase inhibition capacity of the extracts (Fig. 2B). For the AEP, the rise in extraction pH from 7.0 to 9.0 resulted in a 65% decrease in lipase inhibition. Such trend was also observed for polyphenolic extractability. As the extraction pH increased from 7.0 to 9.0, TPC values decreased from 10160 to 7666 mg GAE/100g dry extract and the total phenolic concentration, measured by HPLC, decreased from 8912 to 6220 mg/100 g of dry extract (Table 2, Table 3). As previously reviewed, polyphenols are potential sources of alternative treatments for obesity considering their ability to inhibit lipase (Buchholz and Melzig, 2015).

When evaluating the potential impact of enzymatic extraction on the extract's ability to inhibit lipase activity, it was observed that overall, all EAEP extracts exhibited lower lipase inhibition when compared to their respective controls (∼52% reduction for NP and ∼70% reduction for AP) (Fig. 2B), indicating that coffee protein hydrolysates are not efficient at inhibiting lipase activity at the tested concentrations. Indeed, considering previous *in silico* simulation carried out with coffee proteins (Ribeiro et al., 2021), no peptide sequences were identified as lipase inhibitors. Likewise, a pre-treatment with carbohydrase (30 min), followed by NP or AP (30 min), did not affect lipase inhibition (p > 0.05) compared with the use of NP or AP alone for 60 min (Fig. 2B), but it reduced lipase activity compared with their controls (AEP at pH 7.0 and 9.0). Similar to the AEP extracts, higher lipase inhibition was observed for the EAEP extracts obtained at 7.0 compared with those obtained at pH 9.0. The lipase inhibition values of the extracts generated by NP were consistently higher than those of the extracts generated by AP, which is consistent with their antioxidant activity by ORAC (ORAC ∼ 1.05 *vs.* 0.74 mmol Trolox equivalent/g dried extract for NP and AP, respectively) and chlorogenic acid content (Table 3). Therefore, in the present work, the lipase inhibitory activity may be attributed mainly to the chlorogenic acid present in the coffee extracts (Table 3).

The potential of green coffee bean flour to inhibit lipase when pure (EC_50_ = 2.52 mg/mL) or added to bread formulations (EC_50_ - 2.02–5.4 mg/mL) has been evaluated previously (Jakubczyk et al., 2018). Considering that the control bread and the wheat flour did not present any lipase inhibitory activity properties, the authors attributed the observed effect only to the green coffee bean flour in the bread formulation. Additionally, both green coffee extracts prepared in acidified water or acetonitrile and decaffeinated green coffee beans (purchase from a local market) have demonstrated effectiveness in inhibiting pancreatic lipase, with inhibition ranging from 11.8 to 61.5% (Almoosawi et al., 2010) and an IC_50_ value of 1.98 mg/mL (Narita et al., 2012). According to the latter study, the inhibition has been mainly attributed to the presence of caffeic acid (IC_50_ = 0.25–1.25 mg/mL) and ferulic acid derivatives (IC_50_ > 2.95 mg/mL). The results presented herein (lipase inhibition varying from 9 to 66% at 7.5 mg/mL extract concentration) agree with the lipase inhibition range found in the literature.

### 3.5 α-glucosidase inhibitory activity

α-glucosidase inhibition is one of the therapeutic approaches for preventing type II diabetes due to the retardation of carbohydrate digestion (Fasihi et al., 2019). α-glucosidase is an oligosaccharide-hydrolase enzyme secreted from the intestinal chorionic epithelium that can hydrolyze the bond between the anomeric carbon of the glucosyl residue and glucosidic oxygen, producing α-glucose (Chiba, 1997). Some synthetic inhibitors have been used to treat diabetes mellitus such as acarbose and miglitol. However, some concerns have been raised due to gastrointestinal disturbances and their potential toxic properties. Consequently, the discovery of safe drugs with fewer or no side effects, especially those sourced from natural products, is important (Zengin et al., 2020). In this context, the ability of green coffee extracts to inhibit α-glucosidase activity was evaluated (Fig. 2C).

Although all extracts demonstrated some level of activity at inhibiting α-glucosidase, the maximum inhibition was limited to 14% (Fig. 2C). Increasing the pH from 7 to 9 did not affect the inhibition activity of the AEP extracts. Moreover, only the treatment using alkaline protease for 30 min (AP 30 min) increased the inhibitory activity of the extracts when compared to the neutral protease (NP-30 min), but the value was similar to that of the control. The low inhibitory activity of the green coffee extracts is in agreement with a previous study that investigated the *in vitro* α-glucosidase inhibitory activity for unroasted and roasted robusta coffee and did not find any inhibitory activity for green coffee extracts (Alongi and Anese, 2018). However, the later study showed a positive correlation between the degree of roast and the inhibition of α-glucosidase. These findings were attributed to structural changes induced by the roasting and the complexity of interactions between bioactive compounds.

The α-glucosidase inhibitory activity for bread fortified with green coffee flour has also been previously investigated (Jakubczyk et al., 2018). Although no inhibitory effects were identified for green coffee beans (GCB) and the wheat flour by itself, all bread formulations containing GCB inhibited α-glucosidase (IC_50_ 0.03–2.03 mg/mL), probably due to interactions between the bread components, which might have influenced the inhibitory activity of the enzyme. In that view, further research is warranted to explore the potential synergetic or antagonistic effects of the numerous compounds released during the extraction processes (AEP vs. EAEP) used for preparing the green coffee extracts in the present study.

As previous *in silico* simulation for coffee proteins has not identified any peptide sequences that could inhibit α-glucosidase (Ribeiro et al., 2021), the minimum activity at blocking the enzyme is likely not related to coffee peptides. Moreover, as no correlation was found between the increase in phenolic content (>TPC) or phenolic composition/concentration of coffee extracts and their α-glucosidase inhibitory activity in the present work or previously reported (Alongi and Anese, 2018), the small inhibitory effect demonstrated by the AEP/EAEP extracts might be due to the complexity of the green coffee extracts, whose bioactive compounds such as polyphenols can undergo structural changes during extraction and undergo interactions that can affect their biological activity.

### 3.6 Antimicrobial activity

Plant secondary metabolites such as alkaloids and polyphenols have been known not only for their antioxidant properties but also for their antibacterial activity (Othman et al., 2019). The bacterial inactivation mechanism can involve several factors, such as changes in permeability of cell membranes or cell wall rigidity, changes induced by hydrogen binding of the phenolic compounds to enzymes, and enzyme inhibition through reactions with sulfhydryl groups on proteins, among others (Othman et al., 2019; Bouarab-Chibane et al., 2019). In addition, more recently, antimicrobial peptides have been accepted as a new class of bioactive compounds derived from proteins that present unique antimicrobial properties. Their action mechanism is related to changes in membrane permeability, pH gradient, membrane potential, and osmotic regulation (Dhayakaran et al., 2016).

The ability of coffee extracts to inhibit microorganisms such as *S. aureus*, *B. cereus*, *E. faecalis*, *L. monocytogenes*, *E. coli,* and *S. typhimurim* was tested. Because *S. aureus* (Gram positive) was significantly inhibited by the extracts in the antimicrobial screening assay compared to the other microorganisms, this strain was followed by an antimicrobial quantitative analysis to calculate the surviving fraction, and *E. coli* (Gram negative) was used for comparison (Fig. 2D).

The antimicrobial activity for the green coffee extracts against *S. aureus* was always higher than for *E. coli*, regardless of the extraction process used to prepare the extracts (Fig. 2D). In fact, Duangjai et al. (2016) has shown that chlorogenic acid and caffeine-rich coffee pulp extracts were effective against *Staphylococcus aureus* and *Escherichia coli,* but the inhibition zone for Gram-positive bacteria was larger than for Gram-negatives. Authors suggested that these differences are due to the phospholipid-rich Gram-negative bacterial outer membrane that acts as a barrier for hydrophobic compounds.

The use of enzymes in the EAEP did influence the antimicrobial activity of the extracts against *E. coli* and *S. aureus* strains in varying ways. While the use of AP did not alter the antimicrobial activity of the extracts against *E. coli* compared with the control (AEP at pH 9.0), the use of NP during the extraction increased the survival rate of both microbes compared with the control (AEP at pH 7.0) (Fig. 2D). Such differences could be attributed to the specific mode of action of the enzymes used, potentially resulting in the release of specific compounds such as polysaccharides and peptides that stimulate microbial growth. Although this aspect was not measured in our current study, it has been previously shown for probiotics (Sales et al., 2020). Further investigation is needed to explore potential mechanisms.

Considering the high polyphenol content of both AEP and EAEP extracts (Table 2, Table 3) and the previous *in silico* analysis for coffee 11S coffee globulin, that showed a high frequency of occurrence (A) for angiotensin I-converting enzyme (ACE) inhibitor peptides (0.44) and low frequency for antibacterial activity (A = 0.004) (Ribeiro et al., 2021), we can infer that the low antimicrobial activity of the coffee extracts (<10^1^ CFU/mL reduction), in the present research, could be primarily associated to the presence of polyphenol compounds and xanthines (e.g. caffeine and trigonelline), as shown in Table 3 (Sales et al., 2020), instead of resulting from peptide activity. As discussed in the literature, polyphenols can act differently against microorganisms and the mechanisms may include complexation with extracellular and soluble proteins or bacterial cell walls, disruption of lipophilic microbial membranes (as found for flavonoids), inhibition of enzymes through reactions with sulfhydryl groups on the proteins, and the inactivation of microbial adhesins (Othman et al., 2019; Cowan, 1999).

To summarize key research findings, Table 4 presents the main observations from each characterization, with a particular focus on the differences between AEP and EAEP treatments.

**Table 4:** Summary of the main findings, highlighting the influence of the different extraction process on the biological activity of coffee extracts.

Biological activity	Main remarks
Total polyphenol content (TPC)	• EAEP extracts presented similar TPC to the respective aqueous extracts. • TPC values were higher at AEP – pH 7.0 compared to AEP – pH 9.0 (polyphenol degradation).
Chlorogenic acid	• Higher at neutral pH, regardless of the process – up to 6335.32 mg/100 g dry extract
Antioxidant activity	• Higher for AEP extracts obtained at pH 7 compared with pH 9.0 • NP – 60 min higher than AP – 60 min due to higher extractability of caffeic acid (130.88 vs. 90.24 mg/100 g dry extract), cinnamic acid (32.46 vs. 23.74 mg/100 g dry extract) and ferulic acid (329.34 vs. 126.54 mg/100 g dry extract)
In vitro digestibility	• High *in vitro* protein digestibility for all coffee extracts (>98%)
ACE inhibitory activity	• Highest for EAEP coffee extracts – up to 85%
Lipase inhibitory activity	• Highest for AEP coffee extracts – up to 66%
α-glucosidase inhibitory activity	• Low for all coffee extracts (≤14%)
Antimicrobial activity	• Poor growth inhibition for all coffee extracts (<10^1^ CFU/mL reduction)

## 4 Conclusion

This study describes the impact of aqueous and enzymatic extraction approaches on the nutritional and biological properties of green coffee extracts. Different biological activities were identified for the extracts and were attributed to both proteins and their hydrolysates, besides phenolic compounds – specially chlorogenic acid. High *in vitro* protein digestibility was observed for all extracts, regardless of the extraction pH and enzyme used, which is likely due to the low molecular weight of the peptides, which facilitate the access of digestive enzymes. Compared to neutral medium, alkaline pH decreased the amounts of some phenolics and, consequently, antioxidant activity of the extracts, probably due to degradation of compounds such as chlorogenic acid, catechin, epicatechin, cinnamic acid, besides caffeine (a xanthine). Although neutral protease achieved higher phenolic extractability than alkaline protease at 60 min, the yield was comparable to that of the control (AEP – pH 7.0), indicating that the enzyme treatment does not substantially improve overall extractability compared to the control. The use of neutral and alkaline proteases in the EAEP resulted in extracts with higher ACE inhibitory activity compared with their respective controls (AEP- pH 7.0 and 9.0), likely related to the phenolic compounds (mainly chlorogenic acid) and smaller peptides resulting from the EAEP process. Conversely, enzymatic extractions (52% reduction for NP and 70% reduction for AP) and alkaline medium (65% decrease) were not efficient at producing extracts with lipase inhibition properties, whose best inhibitory activity was observed for the aqueous extract obtained at neutral pH (AEP - pH 7.0–66%). Low α-glucosidase (<14%) and antimicrobial inhibitory activities were observed. In the last case, a low CFU reduction (<10^1^ CFU/mL reduction) and an increase in survival rate for *E. coli* and *S. aureus* was sometimes noticed, as specific compounds can be released and stimulate microbial growth. Overall, AEP and EAEP coffee extracts are suitable for use as enzyme inhibitors primarily in the context of anti-obesity and antihypertensive treatments. In addition to maximizing protein and lipid yields and protein functionality, enzymatic extraction resulted in peptides that contributed to their higher biological activity. Although *in vitro* studies do not fully reflect the *in vivo* metabolism of bioactive compounds, the present research highlights the potential for modulating the composition of green coffee extracts through different sustainable extraction processes (AEP and EAEP), which directly impacts their biological activities and possible applications. Additional research might be valuable to explore *in vivo* validation methods to substantiate the benefits of consuming green coffee extracts in reducing blood pressure and obesity in humans.

## CRediT authorship contribution statement

**Flávia Souza Almeida:** Conceptualization, Methodology, Data curation, Formal analysis, Writing – original draft, Writing – review & editing. **Fernanda Furlan Gonçalves Dias:** Conceptualization, Methodology, Data curation, Writing – review & editing. **Matthew William Ford:** Methodology, Writing – review & editing. **Stanislau Bogusz Junior:** Methodology, Writing – review & editing. **Ana Carla Kawazoe Sato:** Conceptualization, Supervision, Funding acquisition, Writing – review & editing. **Juliana Maria Leite Nobrega de Moura Bell:** Conceptualization, Data curation, Supervision, Project administration, Funding acquisition, Writing – review & editing.

## Funding

This work was funded by the USDA National Institute of Food and Agriculture, Hatch/Multi State project, United States [CA-D-FST-2348-RR] and partially supported by the Coordination for the Improvement of Higher Education Personnel - Brazil (CAPES) – Brazil (Finance code 001), by the National Council for Scientific and Technological Development (CNPq) – Brazil (Grant number # 141112/2018-2) and by the Sao Paulo Research Foundation - Brazil (FAPESP) (10.13039/501100001807FAPESP # 2018/21987-1 and # 2022/03229-8).

## Declaration of competing interest

The authors declare that they have no known competing financial interests or personal relationships that could have influenced the work reported in this paper.

## Data Availability

Data will be made available on request.
