# Supplementary material for: Exploring the nutritional and biological properties of green coffee extracts: A comparative study of aqueous and enzymatic extraction processes
Source: Curr Res Food Sci. 2024 Oct 29;9:100890. doi: 10.1016/j.crfs.2024.100890 (PMC11615923; doi:10.1016/j.crfs.2024.100890)
Supplement: Multimedia component 1 [file mmc1.docx]

**Supplementary material**

**Table 1S:** Composition of the digestive fluids: Simulated Salivary Fluid (SSF), Simulated Gastric Fluid (SGF) and Simulated Intestinal Fluid (SIF).

| **SSF** | Final concentration (mg/mL) | Final pH |
| --- | --- | --- |
| Alpha-amylase | 75 U/mL | 7 |
| Mucin | 1.0 |  |
| NaCl | 0.117 |  |
| KCl | 0.149 |  |
| NaHCO_3_ | 2.1 |  |
| **SGF** | Final concentration (mg/mL) | Final pH |
| Pepsin | 2000 U/mL |  |
| Gastric Mucin | 1.5 | 3 |
| NaCl | 8.8 |  |
| **SIF** | Final concentration (mg/mL) | Final pH |
| Pancreatin | 800 U/mL |  |
| Bile extract | 10.0 | 7 |
| NaHCO_3_ | 16.8 |  |

**
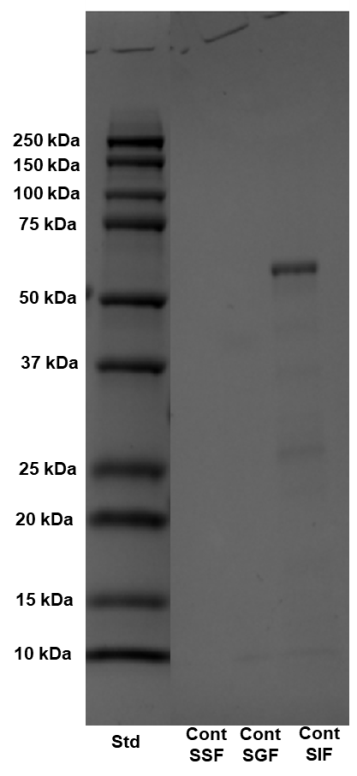
**

**Fig. 1S:** SDS-PAGE control gel for digestion simulation containing only the digestive fluids and enzymes. SSF represents the simulated salivary fluid; SSG the simulated gastric fluid, and SIF the simulated intestinal fluid, respectively.

**Table 2S** – Validation parameters for linear range, limits of detection (LOD), and limits of quantification (LOQ) for caffeine and phenolic compounds in green coffee extracts by HPLC-DAD.

| **Compound** | **Slope** | **Intercept** | **R^2^** | **LOD*** | **LOQ*** |
| --- | --- | --- | --- | --- | --- |
| Gallic acid | 153125 | + 150260 | 0.9981 | 0.36 | 1.20 |
| Chlorogenic acid | 138336 | + 80573 | 0.9994 | 0.40 | 1.33 |
| Quercetin | 50318x | + 80220 | 0.9997 | 1.10 | 2.65 |
| Caffeic acid | 101223 | + 30657 | 0.9989 | 0.54 | 1.82 |
| (-) Epicatechin | 88058 | - 194296 | 0.9982 | 0.63 | 2.09 |
| Cinnamic acid | 151638 | + 16406 | 0.9997 | 0.37 | 1.21 |
| Vanillin | 95431 | + 50205 | 0.9997 | 0.58 | 1.93 |
| m-Cumaric acid | 133487 | + 212885 | 0.9976 | 0.41 | 1.38 |
| Ferulic acid | 97354 | + 115693 | 0.9965 | 0.57 | 1.89 |
| o-Cumaric acid | 135959 | + 4149.6 | 0.9994 | 0.41 | 1.35 |
| Caffeine | 92963 | - 182301 | 0.9987 | 0.59 | 1.98 |
| Resveratrol | 135391 | + 64580 | 0.9986 | 0.46 | 1.36 |
| Catechin | 60221 | + 118677 | 0.9965 | 0.92 | 2.98 |

* LOD e LOQ expressed in mg·mL^-1^


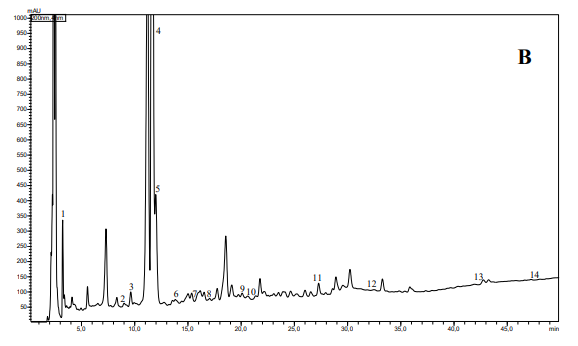

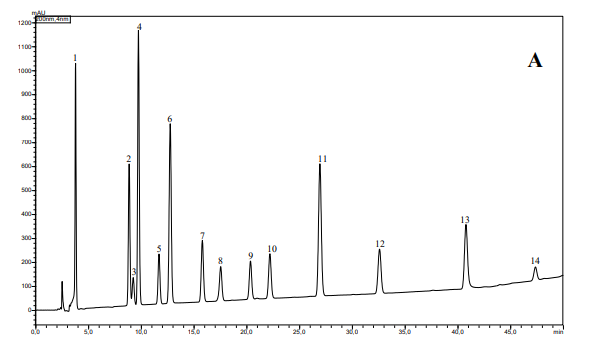


**Fig. 2S:** Chromatograms of the phenolic compounds (A = standards and B = green coffee sample). 1 = gallic acid, 2= quercetin, 3= caffeic acid, 4 = chlorogenic acid, 5 = epicatechin, 6 = cinnamic acid, 7 = vanillin, 8 = m-cumaric acid, 9 = ferulic acid, 10 = o-cumaric acid, 11 = caffeine, 12 = resveratrol, 13 = catechin, 14 = p-cumaric acid.


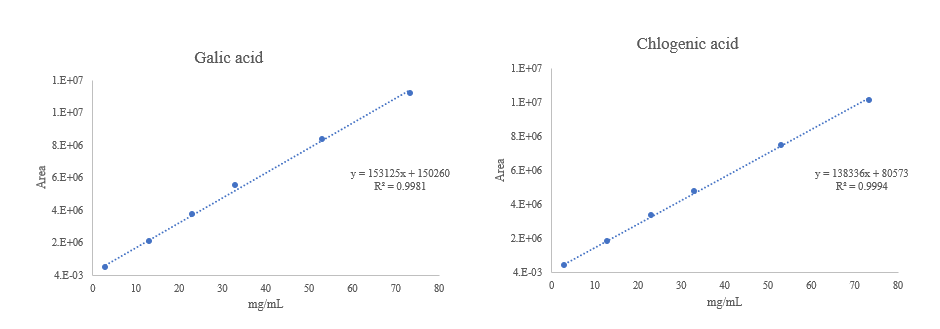


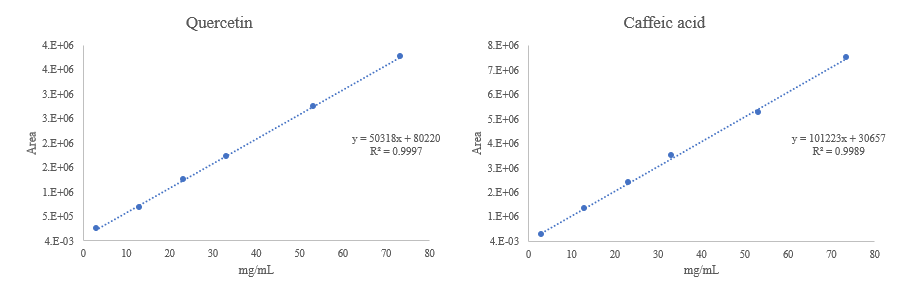

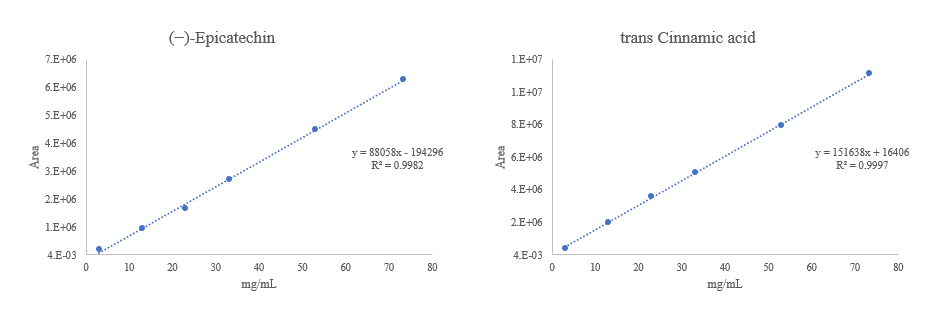


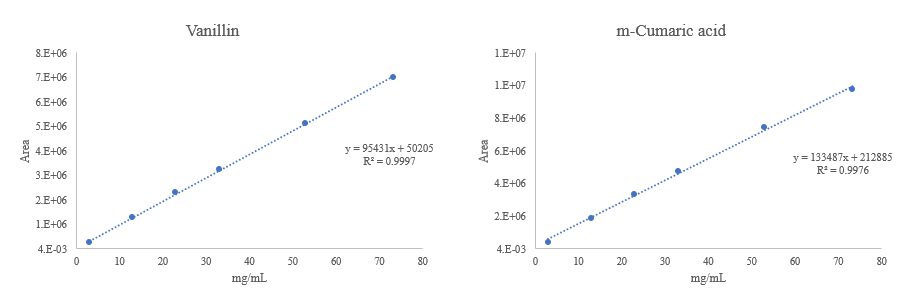


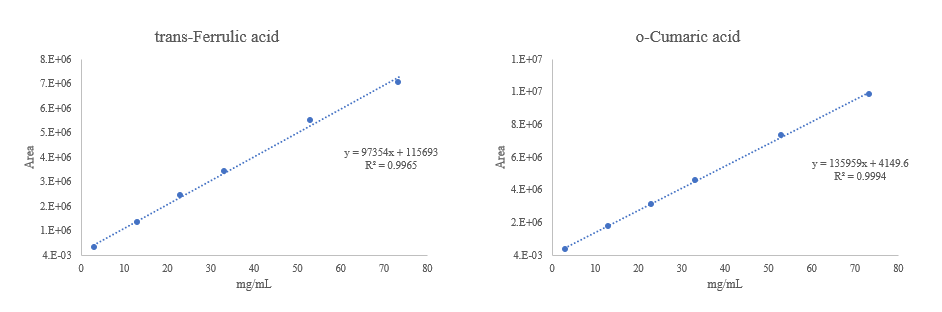


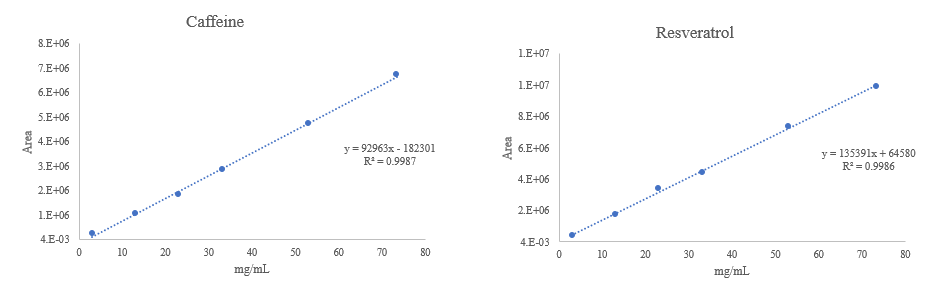

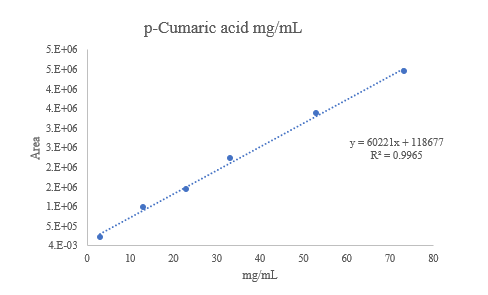


**Fig. 3S –** Calibration curves for the phenolic compounds, i. e., gallic acid, chlorogenic acid, quercetin, caffeic acid, (-) epicatechin, cinnamic acid, vanillin, m-coumaric acid, trans-ferulic acid, o-coumaric acid, resveratrol, p-coumaric acid, and caffeine.


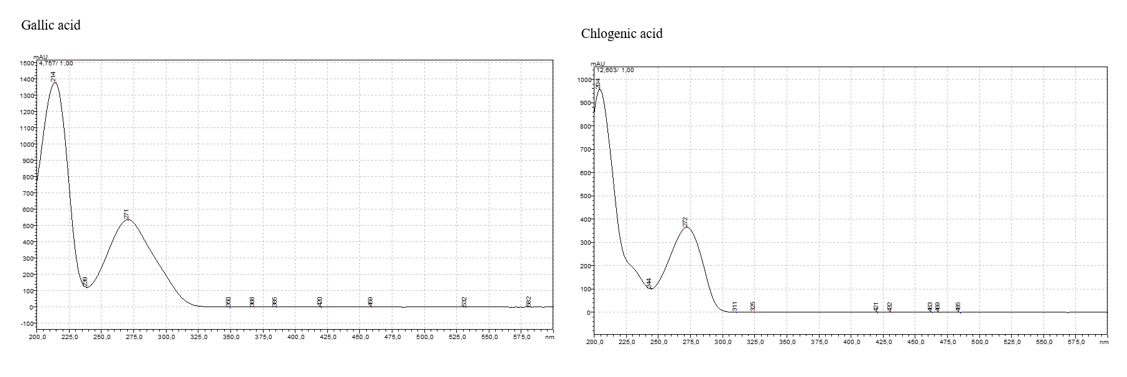

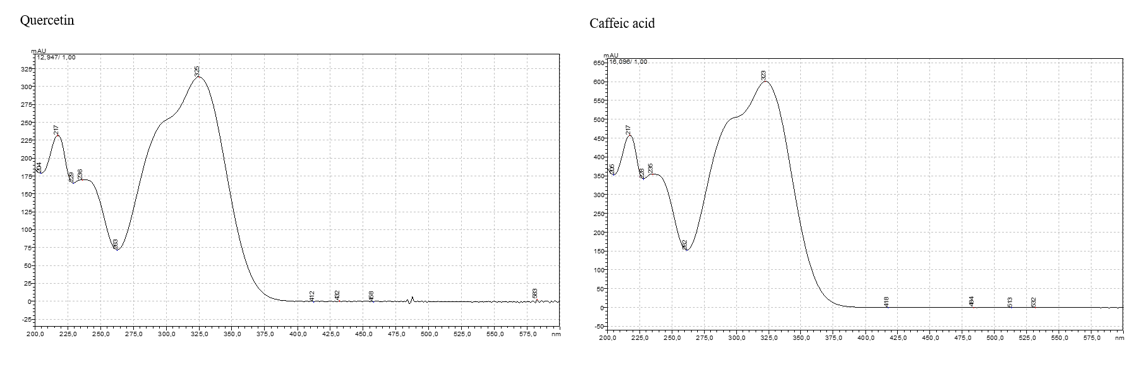

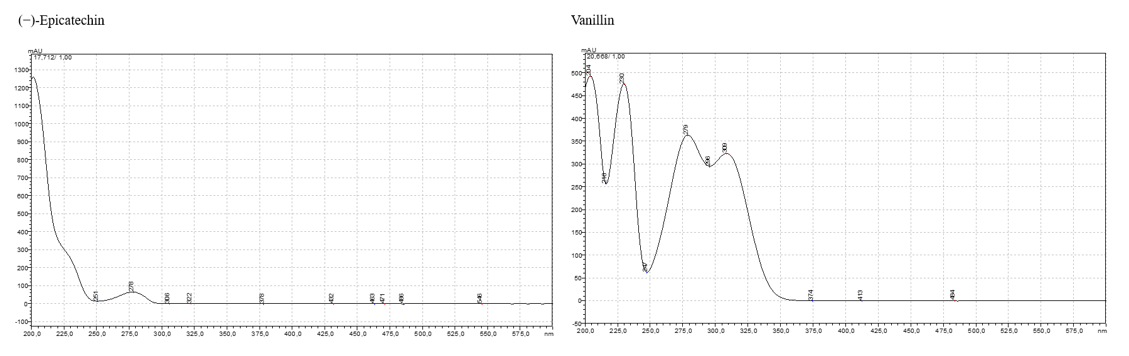


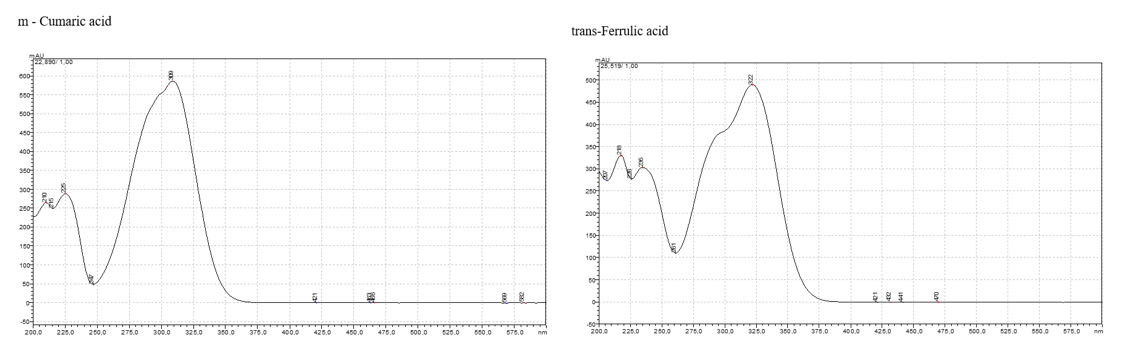

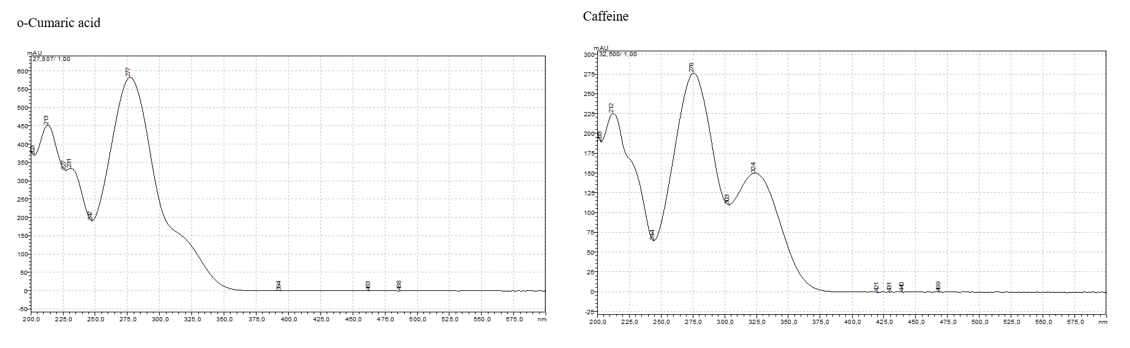

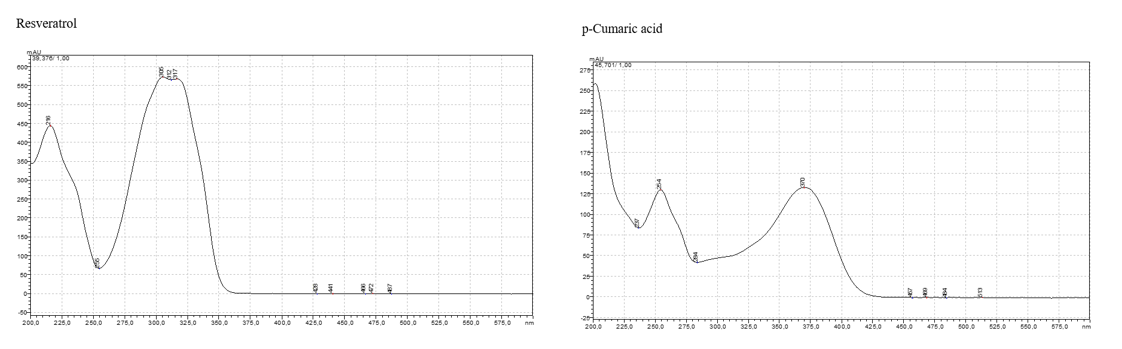


**Fig. 4S** – UV-vis spectra of the identified and quantified phenolic compound standards in green coffee extract samples.
